# Notch Ligand Binding Assay Using Flow Cytometry

**DOI:** 10.21769/BioProtoc.2637

**Published:** 2017-12-05

**Authors:** Shweta Varshney, Pamela Stanley

**Affiliations:** Department of Cell Biology, Albert Einstein College of Medicine, New York, USA

**Keywords:** Notch ligand binding assay, DLL1, DLL4, JAG1, JAG2, Fc-tag, Flow cytometry

## Abstract

Notch signaling is an evolutionary conserved signaling pathway that plays an indispensable role during development, and in the maintenance of homeostatic processes, in a wide variety of tissues (Kopan, 2012; Hori *et al.,* 2013). The multifaceted roles of Notch signaling are stringently regulated at different levels. One of the most important aspects of regulation is the binding of different Notch ligands to each Notch receptor (NOTCH1-NOTCH4). Canonical ligands Delta or Serrate (in *Drosophila*), and Delta-like (DLL1 and DLL4) or Jagged (JAG1 and JAG2) (in mammals), are transmembrane glycoproteins. Ligands expressed on one cell bind to Notch receptors on an adjacent cell to induce Notch signaling. Glycosylation of Notch receptor extracellular domain by O-fucose and O-GlcNAc glycans is well established as critical regulators for Notch signaling strength (Stanley and Okajima, 2010; Haltom and Jafar-Nejad, 2015; Sawaguchi *et al.,* 2017). In order to characterize Notch ligand binding to Notch receptors in isolated cells, we utilize Notch ligand extracellular domains tagged at the C-terminus by a human Fc domain, and determine binding of fluorescent anti-Fc antibody by flow cytometry.

## Background

**Figure 1:**
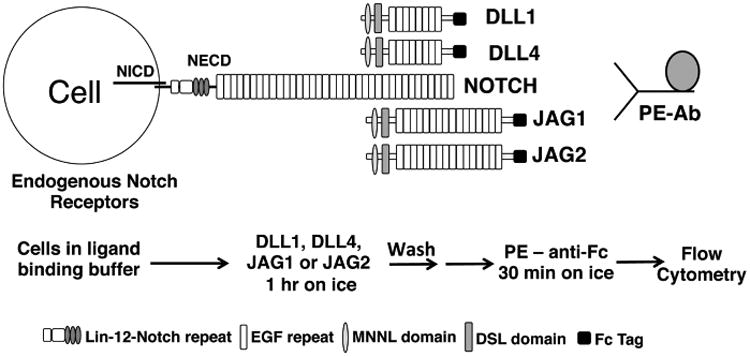
Diagram of the Notch ligand binding assay Notch receptors expressed on the cell surface have an extracellular domain (NECD) comprised of 29-36 N-terminal EGF like repeats followed by 3 Lin-12 Notch repeats. Cell surface expression is confirmed using anti-NECD antibodies. NECD is non-covalently attached to the intracellular domain (NICD) which gets released from the cell membrane and translocates to the nucleus upon proteolysis following ligand binding. Notch ligands Delta-like (DLL1 and DLL4) and Jagged (JAG1 and JAG2) extracellular domains (ECD) comprise a Module at the N-terminus of Notch Ligand (MNNL) motif, followed by a Delta-Serrate-LAG2 (DSL) domain, followed by 6-16 EGF repeats. For this assay, the C-terminus of Notch ligand ECD is linked to a human Fc-tag which is recognized by a fluorescently-labeled secondary antibody (PE-Ab). Ligand binding buffer must contain calcium for Notch ligand binding to occur. Chelation of calcium is used as a control for the specificity of ligand binding.

Cell proliferation, differentiation, and apoptosis are well known to be regulated by Notch signaling. Aberrant changes in Notch signaling are related to diverse disorders, giving rise to a range of developmental and adult diseases (Bray, 2016). The canonical Notch signaling pathway in mammals is initiated by the binding of Notch ligands Delta or Jagged to the extracellular domain of Notch receptors (NECD), expressed on opposing cells. Receptor-ligand binding initiates two sequential proteolytic cleavages, resulting in the release of the Notch intracellular domain (NICD). Released NICD complexes with the transcriptional repressor CSL (CBF-1/Suppressor-of-hairless/Lag-1), also termed recombination signal binding protein for immunoglobulin kappa J region (RBPjk), and the co-activator Mastermind (MAML), activate Notch target genes. The binding of Notch receptors to different ligands results in distinct consequences (Benedito *et al.,* 2009; Bray, 2016). For example, the maintenance of hematopoietic stem cells is regulated by low strength JAG1-mediated Notch signaling, whereas arterial cell fate is determined by high strength DLL4-mediated Notch signaling (Gama-Norton *et al.,* 2015). The addition of N-acetylglucosamine (GlcNAc) to the O-fucose on epidermal growth factor (EGF) repeats of the NECD by a Fringe glycosyltransferase generally enhances signaling by Notch receptors induced by Delta-like ligands DLL1 and DLL4, while reducing signaling induced by Jagged ligands JAG1 and JAG2 (Bruckner *et al.,* 2000; Moloney *et al.,* 2000; Yang *et al.,* 2005; Kovall *et al.,* 2017). Recent structural studies have revealed molecular interactions between O-glycans on a Notch1 fragment including EGF repeats 8-13, and soluble ligands DLL4 (Luca *et al.,* 2015) and JAG1 (Luca *et al.,* 2017). Notch ligand EGF repeats are also modified with O-glycans but mutant ligands lacking O-glycans largely remain functional (Muller *et al*., 2014; Serth *et al*., 2015). The protocol described below is a method of determining the relative binding of soluble Notch ligand ECDs to the ECD of endogenous or introduced Notch receptors (Figure 1).

## Materials and Reagents

1. Pipette tips (USA Scientific, catalog numbers: 111 0 -3000, 111 0 -1000, 1111 -2021)
2. T75 flask (Corning, Falcon^®^, catalog number: 353136) or 100 mm TC-treated Tissue Culture Dish (Corning, Falcon^®^, catalog number: 353003)
3. 15 ml Falcon tubes (Corning, Falcon^®^, catalog number: 352099)
4. 1.5 ml Eppendorf tubes (USA Scientific, catalog number: 1615-5500)
5. Aluminum foil (Fisher Scientific, catalog number: 01-213-104)
6. 5 ml polystyrene round-bottom tube with cell strainer cap (Corning, Falcon^®^, catalog number: 352235)
7. Syringe derived filtered units, 0.22 μm (Merck, catalog number: SLGV013SL)
8. Amicon Centrifugal filter units, 30K (Merck, catalog number: UFC503024)
9. Enzyme-free cell dissociation solution (Merck, catalog number: S-014-B)
10. Alpha MEM medium (Thermo Fisher Scientific, Gibco™, catalog number: 11900073)
11. Fetal bovine serum (FBS) (Gemini Bio-Products, catalog number: 100-106)
12. Fc Receptor (FcR) block-purified rat-anti-mouse CD16/CD32, clone2.4G2 (mouse BD Fc block) (BD, BD Biosciences, catalog number: 553141)
13. Notch ligands: DLL1, DLL4, JAG1 and JAG2 ECD with a human Fc domain tag at the C-terminus produced from HEK293T cells stably expressing ligand-Fc constructs as described previously (Stahl *et al* ., 2008). Alternatively, soluble, Fc-tagged Notch ligands can be purchased from R&D Systems:
  DLL1 (R&D Systems, catalog number: 5026-DL-050)
  JAG1 (R&D Systems, catalog number: 599-JG-100)
  JAG2 (R&D Systems, catalog number: 4748-JG-050)
  DLL4 (Thermo Fisher Scientific, catalog number: 10171H02H25)
14. Secondary antibody: R-Phycoerythrin (PE) AffiniPure F(ab')_2_ fragment goat-anti-human IgG, Fcγ fragment-specific (Jackson ImmunoResearch, catalog number: 109-116-170)
15. Sodium chloride (Fisher Scientific, catalog number: S271)
16. Sodium phosphate dibasic (Sigma-Aldrich, catalog number: S3264)
17. Potassium phosphate monobasic (Fisher Scientific, catalog number: P285)
18. Potassium chloride (Fisher Scientific, catalog number: P217)
19. Magnesium chloride hexahydrate (Fisher Scientific, catalog number: M33)
20. Calcium chloride dihydrate (Sigma-Aldrich, catalog number: C3881)
21. Hanks' balanced salt solution (HBSS) (Mediatech, catalog number: 55-022-PB)
22. Bovine serum albumin (BSA) (Gemini Bio-Products, catalog number: 700-100P)
23. Sodium azide (Fisher Scientific, catalog number: BP922I-500)
24. 16% paraformaldehyde (PFA) in aqueous solution (Electron Microscopy Sciences, catalog number: 15710)
25. Pro293a™ (Lonza, catalog number: 12-764Q)
26. PBS with cations, pH 7.2-7.4 (see Recipes)
27. Ligand binding buffer (LBB), pH 7.2-7.4 (see Recipes)
28. 4% PFA in PBS (see Recipes)
29. Soluble Notch ECD ligand with Fc tag (see Recipes)

## Equipment

1. Pipettes (Mettler-Toledo, Rainin, catalog numbers: 17008653, 17008650, 17008649; Thermo Fisher Scientific, Thermo Scientific™, catalog number: 4641070N)
2. Benchtop centrifuge (GMI, IEC, model: HN-SII)
3. Benchtop centrifuge (Eppendorf, model: 5417 C)
4. Coulter Particle Counter (Beckman Coulter, model: Z1 Series)
5. Flow cytometer (Cytek Biosciences, model: D×P 10)

## Software

1. Acquisition Software: FlowJo CE 7.5.110.7
2. Analysis Software: FlowJo version 10.3.0.Beta3

## Procedure

1. For adherent cells
  - Remove culture medium and wash the cell layer once with 5 ml of cold PBS (with cations;see Recipes) at room temperature (RT).
  - Remove PBS and add 1 ml of enzyme-free dissociation reagent per T75 flask or 10 cm dish to dissociate the cells at RT.
  - Transfer the flask or dish to 37 °C. After 1 min (minute), check if cells have started to detach.If not, keep them at 37 °C until detachment is obvious.
  - Vigorously tap the sides of the flask or dish to dissociate the cells.
  - After most cells have detached, re-suspend the cells in 9 ml of medium containing 10% FBS to obtain a single cell suspension.
  - Adherent cells that are difficult to dissociate using enzyme-free dissociation reagent, the cells can be scraped off the flask or dish and resuspended as a single cell suspension in medium containing 10% FBS. Be careful allow clumped cells to settle.
2. For single cell suspension obtained above and for cells growing in suspension
  - Count the cells–need at least 2 × 10^6^ cells per ligand per replicate, if using 0.5 × 10^6^ cellsper reaction (0.5 × 10^6^ cells: unstained cells to set the flow cytometer, 0.5 × 10^6^ cells fornegative control, 10^6^ cells for test samples [Notch ligand-Fc]).
  - Centrifuge the required cell volume in a 15 ml Falcon tube at 115 *× g* (1,000 rpm) for 10 min in a benchtop centrifuge (GMI, IEC, model: HN-SII) at RT.
  - Aspirate the supernatant and wash the cell pellet in 10 ml ligand LBB (see Recipes). Be careful not to aspirate the cell pellet.
3. Fix cells in 4% PFA
  - Centrifuge cells as above and aspirate supernatant.
  - Add 1 ml of 4% PFA per 10^7^ cells to the cell pellet and gently resuspend by vortexing in brief spurts in 4% PFA (see Recipes).
  - Incubate cells in 4% PFA for 10 min at RT.
  - Centrifuge at 115 *× g* (1,000 rpm) for 10 min and discard supernatant.
  - Wash the cells twice more with 10 ml LBB.
  - Resuspend cells to a final concentration of 10^6^ cells/ml in LBB.
  - Fixed cells can be stored at 4 °C for at least a month. The advantage of using fixed cells is that endocytosis of membrane receptors cannot occur, different cell types can be prepared and fixed on different days, and the ligand binding assay can be performed on all samples with each Notch ligand-Fc on the same day in one experiment, thereby reducing variation between samples.
  - Unfixed cells can also be used for Notch ligand binding experiments. However, the cells should be used fresh from exponentially growing cultures at 37 °C, and washed in cold PBS, prior to assaying binding at 4 °C. It is important that endocytosis of Notch receptors is prevented and sodium azide at 0.05% can be included in the binding assay for that purpose. Cells that have a damaged plasma membrane can be identified using dyes like 7-amino actinomycin D (7-AAD), Hoechst 33342 and 4,6-diamidino-2-phenylindole (DAPI) in LBB added to cells just prior to flow cytometry, and subsequently gated out of the cells to be analyzed.
4. Experimental design
  Label 1.5 ml Eppendorf tubes and aliquot a fixed number of cells based on the following experimental design.
  - a. Controls for the experiment:
    - Unstained cells for background fluorescence: A mixture of equal numbers of each cell type to be assayed is aliquoted into a 1.5 ml Eppendorf tube so the final cell number is 0.5-1.0 × 10^6^ cells in LBB. Mix briefly by vortexing. This sample is used to establish parameters in the flow cytometer.
    - Negative controls-Fc tag alone or secondary antibody alone: Take an aliquot with 0.5-1.0 × 10^6^ cells in LBB into a 1.5 ml Eppendorf tube for subsequent incubation with control-Fc or secondary antibody alone. A negative control is set up for each cell type.
  - Test samples: Different Notch ligands tagged with Fc
    For each Notch ligand-Fc, aliquot 0.5-1.0 × 10^6^ cells in LBB into separate 1.5 ml Eppendorf tubes.
5. Centrifuge cells aliquoted above at 420 *× g* (2,000 rpm) in a benchtop centrifuge (Eppendorf, model: 5417 C) for 5 min at 4 °C, and discard the supernatant.
6. Wash cells with 1 ml ice cold LBB.
7. Repeat the wash once and discard the supernatant.
8. Add 50 μl FcR block (diluted 1:50 in LBB) to all cells, except the unstained control cells, which receive 50 μl LBB, and gently vortex.
9. Incubate for 15 min on ice. Do not wash.
10. Add 100 μl Notch ligand-Fc or Fc-tag alone (100-1,000 ng in 100 μl LBB). To unstained cells, or cells that will receive secondary antibody alone, add 100 μl LBB. Gently vortex each tube to mix.
11. Incubate for 1 h on ice with intermittent mixing by hand every 15 min, or at 4 °C with rotation.
12. Centrifuge at 420 *× g* (2,000 rpm) for 5 min at RT, and discard the supernatant.
13. Wash cells with 1 ml ice cold LBB.
14. Repeat wash once and discard the supernatant.
15. Add 100 μl secondary anti-Fc antibody (1:100 in LBB) to all cell pellets, except the unstained cell pellet, and gently vortex to resuspend.
16. Incubate for 30 min on ice, or at 4 °C with rotation. Cover tubes with aluminum foil to protect the samples from light.
17. Centrifuge at 420 *× g* (2,000 rpm) for 5 min at RT, and discard the supernatant.
18. Wash the cells with 1 ml ice cold LBB.
19. Repeat wash once and discard supernatant.
20. Add 250-500 μl of ice cold LBB to each cell pellet, mix and pass through the strainer cap of a 5 ml polystyrene round-bottom tube. This removes clumped cells immediately prior to flow cytometry. It is essential to have a single cell suspension prior to proceeding with flow cytometry–a) Clumped cells interfere with the analysis. b) Clumped cells can clog the flow cytometer.
21. Proceed to flow cytometry. Care should be taken that samples are exposed to minimum light.

## Data analysis

**Figure 2:**
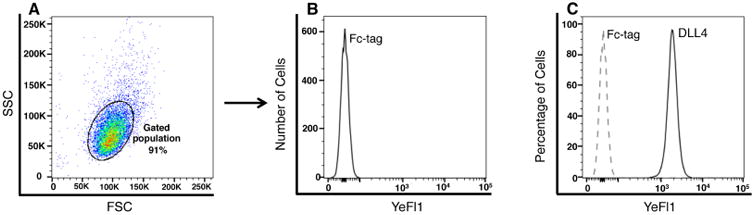
Flow cytometer profile of DLL4-Fc ligand binding to Chinese hamster cells (CHO) A. Profile represents gating on the major population of cells in the SSC vs. FSC profile. B. The profile generated on the gated population of cells treated with Fc-tag alone followed by secondary antibody conjugated to the fluorochrome PE. C. The profile of CHO cells binding Fc-tag alone (dotted line) overlaid with the profile of DLL4-Fc binding (solid line).

- Acquiring data on the flow cytometer
  Unstained cells are used to set the parameters of the flow cytometer. In the flow cytometer acquisition software, open two graph profiles:
  1. Side Scatter (SSC) on the x-axis and Forward Scatter (FSC) on the y-axis.
  2. Histogram on the x-axis and YeFL1 channel on the y-axis (channel on the flow cytometer used to detect the fluorescent secondary antibody).
  3. Set the voltage for SSC vs. FSC such that the majority of the cell population is in the middle of the SSC vs. FSC graph (Figure 2A). Using unstained cell sample, set the second graph histogram vs. YeFL1 channel to a voltage such that the histogram profile is towards the x-axis (≤ 10^2^). Use the same settings to acquire all experimental samples. The samples treated with Fc-tag alone or secondary antibody alone are recorded first and then the samples treated with ligand-Fc are acquired. Acquire at least 20,000 cells per sample for cultured cells. If cell numbers are limiting, the minimum number acquired could be as low as 3,000. Use either the slow or medium speed on the flow cytometer to acquire samples, and keep it the same for all samples. Care should be taken to avoid using the fast run speed. The run speed is determined by the differential pressure applied to move cells through the laser. A high speed increases the number of cells moving through the laser, leading to an increase in coincident events.
- Analysis of the data
  Open the entire data set as a workspace on the flow cytometer acquisition software–FlowJo version 10.3.0.Beta3. Other versions of the software can also be used. Using the unstained cells–gate on the mass population of cells using the SSC vs. FSC graph, avoiding small and large or clumped cells. The sub-population of cells gated on will be represented separately, below the main profile of the sample. On this major sub-population of cells change the x-axis to histogram and the y-axis to YeFL1 channel (channel on the flow cytometer used to detect the secondary antibody). Apply this gate to all the samples. The profiles of the Fc alone and Notch ligand-Fc can be plotted using layout editor in the acquisition software (Figure 2B). To compare binding in different sample populations, create overlays in the layout editor. To create overlays: drag the population of the first sample from the workspace to the layout editor, and then drag the second sample population on top of the first graph (Figure 2C). Remember to use the same gate for Fc alone/secondary only and Notch ligand-Fc for each cell type. The mean fluorescence intensity (MFI) of the samples can be calculated by using the statistics toolbar and selecting median on the YeFL1 channel. Data replicates are compared by relative MFI ± SEM; significance determined by paired, two-tailed Student's *t*-test n ≥ 3.

## Notes

Notch receptor/Notch ligand binding requires the presence of calcium. In the assay above, LBB contains 1 mM CaCl2, binding does not occur in the presence of a low concentration (5 mM) of metal chelator (EDTA or EGTA) (Stahl *et al*., 2008). This method can be utilized to determine binding parameters for different Notch ligands binding to endogenous or overexpressed Notch receptors present at the surface of any cell type, from cultured to cells isolated from different tissues. This protocol has been tested for adherent cell as well as cells growing in suspension. For example, adherent mouse embryonic stem cells (Stahl *et al.*, 2008) and suspension-grown CHO cells (Hou *et al*., 2012, Sawaguchi *et al*., 2016). Cells were always grown at 37 °C in alpha MEM medium containing 10% FBS unless otherwise stated. Ligand binding for each ligand was typically performed in 100 μl of LBB containing 0.5 × 10^6^ cells. Intracellular NECD can be detected by the same method after cell permeabilization. The method is highly sensitive, reliable and reproducible.

## Recipes

1. PBS with cations, pH 7.2-7.4
  137.93 mM sodium chloride
  8.05 mM sodium phosphate dibasic
  1.47 mM potassium phosphate monobasic
  2.67 mM potassium chloride
  0.49 mM magnesium chloride (hexahydrate)
  0.90 mM calcium chloride (anhydride)
2. Ligand binding buffer (LBB), pH 7.2-7.4
  HBSS
  1% BSA
  1 mM CaCl2
  0.05% sodium azide
3. 4% PFA in PBS
  Dilute 16% PFA (1 in 4 with PBS containing cations)
4. Soluble Notch ECD ligand with Fc tag
  - Briefly, HEK 293T cells stably expressing a construct for Notch ligand with Fc tag (Yang *et al*., 2005) were grown to 90% confluence before the medium was changed to serum-free Pro293a medium with glutamine
  - After 72 h, conditioned medium containing secreted Fc-tagged ligand is carefully collected trying not to disturb the cell monolayer. The medium is filtered through a 0.22 μm syringe filter and the filtrate is concentrated using an Amicon Ultra 15 centrifugal unit with 30 Kd molecular weight cutoff
  - The concentration of the Fc-tagged ligand is determined using Western blot analysis of titrated Notch ligand compared with known amounts of IgG as described (Stahl *et al*., 2008;Hou *et al*., 2012)
